# An Intact *Brachyury* Function Is Necessary to Prevent Spurious Axial Development in *Xenopus laevis*

**DOI:** 10.1371/journal.pone.0054777

**Published:** 2013-01-24

**Authors:** Cecilia E. Aguirre, Sabrina Murgan, Andrés E. Carrasco, Silvia L. López

**Affiliations:** 1 Laboratorio de Embriología Molecular, Instituto de Biología Celular y Neurociencia ‘‘Prof. E. De Robertis’’ (UBA-CONICET), Facultad de Medicina, Universidad de Buenos Aires, Ciudad Autónoma de Buenos Aires, Argentina; Ecole Normale Supérieure de Lyon, France

## Abstract

We have previously shown that the member of the HES family *hairy2* induces the ectopic expression of dorsal markers when it is overexpressed in the ventral side of *Xenopus* embryos. Intriguingly, *hairy2* represses the mesoderm transcription factor *brachyury* (*bra*) throughout its domain in the marginal zone. Here we show that in early gastrula, *bra* and *hairy2* are expressed in complementary domains. Overexpression of *bra* repressed *hairy2.* Interference of *bra* function with a dominant-negative construct expanded the *hairy2* domain and, like *hairy2* overexpression, promoted ectopic expression of dorsal axial markers in the ventral side and induced secondary axes without head and notochord. *Hairy2* depletion rescued the ectopic dorsal development induced by interference of *bra* function. We concluded that an intact *bra* function is necessary to exclude *hairy2* expression from the non-organiser field, to impede the ectopic specification of dorsal axial fates and the appearance of incomplete secondary axes. This evidence supports a previously unrecognised role for *bra* in maintaining the dorsal fates inhibited in the ventral marginal zone, preventing the appearance of trunk duplications.

## Introduction

The Brachyury or T mutation was first described in 1927 and the mouse gene was isolated in 1990. Notochord differentiation was severely impaired in homozygous mutant embryos, which lacked mesoderm posterior to the 7th somite [1]–[4]. *Brachyury (bra)* is highly conserved in terms of sequences and expression patterns between vertebrates [5]–[6]. Unlike the single gene in higher vertebrates, teleosts and amphibians contain multiple copies of *bra*. The allotetraploid genome in *Xenopus laevis* resulted from the hybridisation of two similar but nonidentical ones. Thus, this species contains the homeologs (also known as allo- or pseudoalleles) *Xlbra-a* and *Xlbra-b* (which were originally named *bra* and *bra2),* and in addition, the separate gene *bra3* [5] [7]–[10]. Most of the analysis reported in the literature was performed with *Xlbra-a* (*bra*).

The T-box DNA-binding protein encoded by *bra* was characterised as a transcriptional activator of mesoderm specific genes [11]–[15]. *Bra* is one of the first genes known to directly respond to mesodermal inducers and is only activated at intermediate Activin concentrations, which induce posterolateral and chordal mesoderm, while the high ones induce dorsoanterior organiser mesoderm but are unable to activate *bra* [5] [16]–[19].

In *Xenopus*, *bra* expression begins at mid-blastula transition. Although it is initially expressed in a widespread fashion, when gastrulation begins the transcripts are detected only in the marginal zone (MZ), in the prospective mesoderm. By the end of gastrulation, they only persist in the notochord and in the circumblastoporal mesoderm [5] [20]–[22]. In ectodermal explants, *bra* is sufficient to specify posterior mesoderm, including somites, but it requires at least the transcription factor *foxA4a* or the BMP antagonist noggin to promote notochord development [5] [23]–[26].

Loss-of-function experiments in *Xenopus* with an interfering *bra* construct (*braEnR*), in which its transcription activation domain was replaced with the repressor domain of *Drosophila engrailed* (EnR), resulted in loss of posterior structures, indicating that *bra* is essential for posterior mesoderm development [13]. *Bra* promotes the convergence-extension movements that elongate the notochord and is necessary but not sufficient for its differentiation [13] [27]–[28].

*Xenopus laevis* has two homeologs of *hairy2,* a member of the HES family of transcription factors [10] [29]–[30]. When overexpressed, both homeologs repress *bra* at gastrula stage [31]–[32], but at the same time, they induce the ectopic expression of organiser-specific genes on the ventral side [31] [33]. Although the effects of *hairy2a* on later development were not previously studied, *hairy2b* overexpression was able to induce an incomplete secondary axis [33]. The knock-down of *hairy2a* increased *bra* expression in the gastrula organiser, and an anterior expansion of the notochordal *bra* domain was observed in neurulae depleted from *hairy2b* [31] [33], indicating that *hairy2* regulates *bra* expression on the dorsal midline (DML) structures or their precursors in the organiser.

*Hairy2* belongs to the family of bHLH-Orange transcriptional repressors [29] [34]. It is expressed in the prospective ectoderm in blastulae, in the deep layer of the Spemann’s organiser (SO) and the notoplate during gastrulation, and later, in the prechordal mesoderm (PM), the floor plate (FP) and the neural crest cells (NCC) [30]–[31] [35]–[36]. Its dynamic expression in the borders of several territories is consistent with the role of *hairy2* in controlling tissue demarcation, for example, favouring the FP fate at the expense of the notochord and maintaining the identity of the anterior PM by repressing specific genes of neighbouring tissues [31] [33] [36]–[38].

The basic body plan of the vertebrates is generated according to early events triggered by the Wnt/β-catenin pathway. After fertilization, the stabilization of the maternal β-catenin protein at the site opposite to the sperm entry point determines the future dorsal side of the embryo, where the Spemann-Mangold organizer will arise [39]–[42]. Dorsal development must also be restricted, and in order to prevent axial duplications, the accumulation of nuclear β-catenin in the ventral side of the embryo is prevented by maternal control mechanisms [41] [43]–[45]. Besides, zygotic mechanisms involving Wnt8 and BMPs are also necessary to maintain the inhibition of dorsal development in ventral locations [46]. In this paper we delve into this issue by showing that repression of *bra* target genes produce strikingly similar phenotypes to those of *hairy2a* overexpression, including the induction of a secondary trunk. The ectopic dorsal development induced after interfering with *bra* function was rescued by *hairy2* depletion. Thus, we conclude that normal development requires an intact *bra* function to exclude the *hairy2* domain from the non-organiser field in order to maintain the inhibition of dorsal axial fates in the ventral marginal zone, thus impeding the formation of spurious incomplete secondary axis.

## Materials and Methods

### Embryological Manipulations, RNA Synthesis, Morpholinos, and Injections

All animal studies in this report followed the rules and protocols approved by the Institutional Review Board for the Care and Use of Laboratory Animals (CICUAL) in the School of Medicine, University of Buenos Aires, Argentina. Albino and wild-type *Xenopus laevis* embryos were obtained using standard methods, staged and fixed with MEMFA [47]; [48]. Synthesis of capped mRNAs and the templates for *bra* and *braEnR* mRNAs were described elsewhere [13] [23] [49]. The full-length *hairy2a* cDNA construct fused to 6 myc-tag epitopes in pCS2+ (MT-*hairy2a*), kindly provided by Dave Turner, was digested with Not I and transcribed with SP6 RNA polymerase. Overexpression experiments for *hairy2a* were performed with MT-*hairy2a* mRNA. The *hairy2a* and *hairy2b* morpholino antisense oligonucleotides (*hairy2a* MO, *hairy2b* MO) used in this study were previously shown to specifically knock-down the corresponding homeolog [31] [33]. As control morpholino (Control MO), we used the standard control oligonucleotide or the random control oligonucleotide 25-N (Gene Tools, LLC, OR, USA). Except for the latter, all MOs were modified with 3′-fluorescein. 1 ng of *nuc-lacZ* mRNA or 10 ng of Dextran Oregon Green 488, MW 10000, anionic lysine fixable (DOG, Molecular Probes, Invitrogen) were co-injected as tracers.

### In situ Hybridisation, Immunodetections, and Histology

The preparation of digoxigenin-labelled antisense RNA probes and the whole-mount in situ hybridisation (ISH) procedure were performed as previously described [50], except that the proteinase K step was omitted. For double ISH for *hairy2* and *bra*, fluorescein-labeled antisense RNA probes were prepared with fluorescein-12-UTP (Amersham). We used either a mix of a *hairy2-*digoxigenin+*bra*-fluorescein probes, or a mix of a *hairy2*-fluorescein+*bra*-digoxigenin probes. After hybridisation, embryos were washed and blocked according to the standard protocol and incubated first with one of the antibodies (1/2000 of anti-digoxigenin-AP or 1/5000 of anti-fluorescein-AP, Fab fragments, Roche). After revealing the first probe with either BCIP or Magenta Phos (Sigma), the corresponding antibody was removed with 0.1M glycine, pH 2.0 with 1% Tween-20 (40 minutes), followed by six 10 minutes washes in MAB buffer and 15 minutes incubation in blocking reagent before adding the second AP-conjugated antibody. In some double ISH we used a *chordin* probe labeled with biotin-16-UTP (Roche), as previously described [51]. The c-myc epitopes were revealed by horseradish peroxidase (HRP) immunohistochemistry or by immunofluorescence as previously described [31] [51]. For histology, 20 µm sections were taken in a microtome and mounted onto gelatine coated slides.

### Ventralisations, Dorsalisations and Cell Death Analysis

Embryos were exposed for 1 minute to UV light (254 nm), or were incubated for 1 hour with 0.1 M LiCl [52]. Whole-mount TUNEL staining was based on a previous protocol [53], with some modifications. After removing the vitelline membrane, embryos were fixed 1 hr in MEMFA, extensively washed in PBS, and stored in ethanol at −20°C. After rehydration in PBS, they were permeabilised in 0.25% Triton-X100/PBS, extensively washed in deionised water, incubated for 1 hour in terminal deoxynucleotidyl transferase (TdT) buffer, and transferred to TdT buffer containing 300 U/ml TdT (Invitrogen) and 1 mM digoxigenin-dUTP (Roche). Incubation was carried out overnight at 23°C. The reaction was stopped in 1 mM EDTA/PBS for 2 hours at 65°C. Embryos were washed 4 times, 30 minutes each in PBS, and twice in 100 mM maleic acid, 150 mM NaCl, pH 7.5, 5 minutes each, at room temperature. Incubation in blocking reagent and staining with NBT/BCIP were performed as for ISH [50]. LysoTracker Red (Lys) staining was based on a previous protocol [54], with some modifications. Lys (Molecular Probes, Invitrogen) was diluted in dimethyl sulfoxide and was added to 0.1× Modified Barth’s Saline (MBS) to yield a final concentration of 75 µM. Embryos were incubated in this solution for 30 minutes at room temperature and then, they were extensively washed with 0.1× MBS and fixed.

## Results

### *Hairy2* and *bra* Progressively Establish Complementary Domains

**Figure 1:**
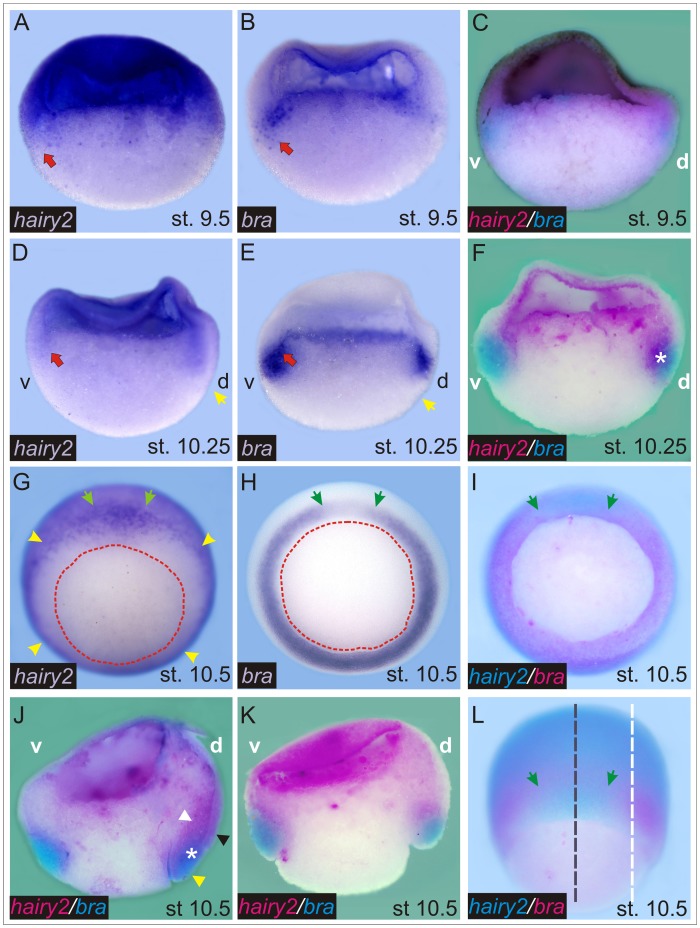
*Bra* and *hairy2* establish complementary expression domains. Distribution of *hairy2* (A,D,G, blue purplish; C,F,J,K, magenta; I,L, turquoise) and *bra* transcripts (B,E,H, blue-purplish; C,F,J,K, turquoise; I,L, magenta). For double ISHs, the name of each probe is written with the colour corresponding to the substrate with which it was revealed. Stage 9.5 (A–C), 10.25 (D–F), and 10.5 (G–L). (A–F,J,K) ISH in bisected embryos. The animal side is oriented to the top. d: dorsal side, v: ventral side. In C–F and J, the embryos were bisected in the sagittal plane, as represented by the broken black line in L. In K, the embryo was cut in a para-sagittal plane, as represented by the broken white line in L. Two halves of the same embryo are shown in the pairs A,B and D,E. The photographs in A,D were flipped 180° to facilitate comparison with the corresponding contralateral hemisection shown in B,E, respectively. Red arrows in A,B,D,E point to the overlapping of *bra* and *hairy2* expression in the MZ. Yellow arrows in D,E mark the incipient blastopore dorsal lip. White asterisks in F,J indicate the overlapping of *hairy2* and *bra* expression on the dorsal side. In J, the white, black and yellow arrowheads point to the prechordal mesoderm, the dorsal NIMZ, and the posterior, pre-involuted axial mesoderm, respectively. (G–I) Whole embryos in vegetal views. Dorsal is oriented to the top. The red broken line demarcates the blastopore. Yellow arrowheads in G point to the NIMZ. The arc between green arrows in G–I,L corresponds to the organiser region, with higher levels of *hairy2* (G,I,L) and lower levels of *bra* expression (H,I,L). (L) Dorsal view of the same embryo shown in I.

We compared the expression of *hairy2* and *bra* during the blastula-gastrula transition. Embryos were bisected in the animal-vegetal plane, and each hemisection was incubated with either a *bra* probe or with a *hairy2* probe that recognises the transcripts of both homeologs [30], and were revealed with NBT+BCIP, which render a strong blue-purplish precipitate. We also performed double ISHs, which were revealed with BCIP and Magenta Phos. Although the reactions with these substrates are less sensitive than those carried out with NBT+BCIP, they were useful to corroborate the results obtained with single ISH. At stage 9.5, *hairy2* transcripts are present in an animal-vegetal gradient, with maximum levels in the animal hemisphere (Fig. 1A). In the MZ, its expression is lower and overlaps with the *bra* territory (Fig. 1A–C). Double ISH in hemisections of pigmented embryos, which allowed to recognise the D–V orientation, showed that at late blastula, *hairy2* expression is higher dorsally, while *bra* expression is higher ventrally and more exclusively expressed in the most vegetal cells of the MZ, while it overlaps with *hairy2* in cells located more animally in the MZ (Fig. 1C).

The domains of these genes are better distinguished in early gastrulae (Fig. 1D–F), when *bra* expression increases considerably in the MZ (Fig. 1E,F). While in the ventral region, their domains are already complementary, in the dorsal one, an overlapping zone still persists (white asterisk, Fig. 1F). Around mid gastrula, *hairy2* is strongly expressed throughout the non-involuting MZ (NIMZ), with highest levels on the dorsal NIMZ (Fig. 1G,I,L) [31], while *bra* demarcates a ring of cells in the involuting MZ (IMZ) in a more vegetal location in relation to *hairy2* (Fig. 1H,I). This ring shows an arc of lower *bra* expression in the organiser region, coincident with the maximum expression of *hairy2* in the dorsal NIMZ (Fig. 1G–I,L). The spatial relationship between *hairy2* and *bra* expression corresponding to this arc can be better appreciated in Fig. 1J, which shows a section of an embryo cut through the sagittal plane, revealing that the strong expression of *hairy2* corresponds to the prechordal mesoderm (white arrowhead) and to the dorsal NIMZ (black arrowhead). On the other hand, the arc of lower *bra* expression corresponds to DML populations which are still segregating and show co-expression of both genes (white asterisk), and to cells of the posterior pre-involuted axial mesoderm, which mainly show *bra* transcripts (yellow arrowhead). Fig. 1K corresponds to a section of an embryo cut through a para-sagittal plane, where the pre-involuted non-axial mesoderm expresses high levels of *bra* in a complementary fashion to the strong *hairy2* expression in the surrounding cells. This complementarity is also clearly observed in the ventral region, where the *bra* domain is larger than in the dorsal side (Fig. 1J,K). In conclusion, the domains of *bra* and *hairy2* become mutually excluded in the MZ, suggesting a possible antagonistic regulation between them.

### *Hairy2* and *bra* are Involved in an Antagonistic Regulation

**Figure 2:**
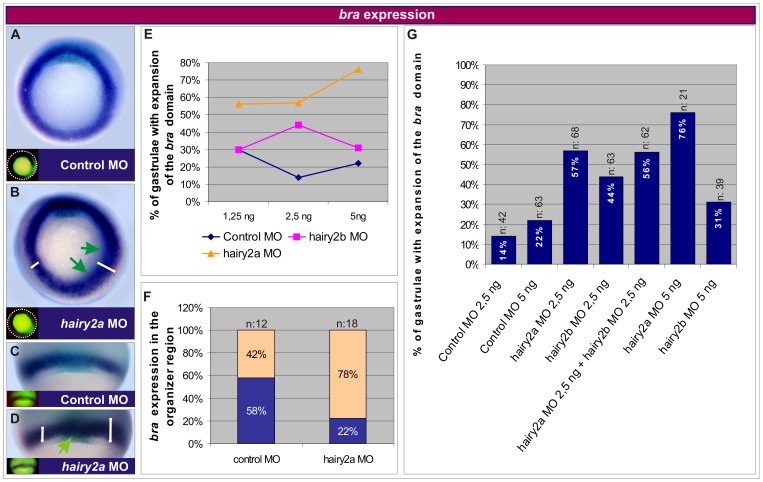
*Hairy2* restricts the *bra* domain. (A–D) Distribution of *bra* transcripts (blue-purplish) at stage 10.5. Turquoise staining corresponds to *chordin* (*chd*) transcripts in the organiser. Embryos were injected into a ventral (A,B,E,G) or dorsal (C,D,E–G) cell at the 4-cell stage with 1.25 ng (C,E), 2.5 ng (A,E–G), or 5 ng (E,G) of control MO; 1.25 ng (D,E), 2.5 ng (B,E–G), or 5 ng (E,G) of *hairy2a* MO; 1.25 ng (E), 2.5 ng (E,G), or 5 ng (E,G) of *hairy2b* MO, or with a mix containing 2.5 ng of *hairy2a* MO +2.5 ng of *hairy2b* MO (G). DOG fluorescence (insets) indicates the injected side. (A,B) Whole embryos in vegetal views. Dorsal is oriented to the top. (C,D) Dorsal views of gastrulae showing *bra* and *chd* expression in the MZ. The green arrows in B,D point to up-regulations or domain expansions of *bra*. White bars in B,D indicate the width of the *bra* domain. (E) Percentage of embryos with *bra* expansion in the MZ. Comparison between different doses of control MO (blue line), *hairy2b* MO (pink line) and *hairy2a* MO (orange line). See Table 1. (F) Percentage of embryos displaying the arc of low *bra* expression that is normally observed in the organiser region (blue) or with an increase of *bra* expression in the organiser (light orange). (G) Percentage of embryos with *bra* expansion in the MZ. Comparison between the simultaneous knock-down of *hairy2a* and *hairy2b* and different doses of control MO, *hairy2a* MO, and *hairy2b* MO. n indicates the total number of injected embryos.

We have previously shown that the injection of *hairy2a* mRNA represses *bra* throughout its domain, while depleting *hairy2a* increases the expression of *bra* within the organiser domain [31]. Now, we extended the analysis to the effects on *bra* expression in the whole MZ by blocking each *hairy2* homeolog with specific MOs against them. At gastrula stage, *hairy2a* MO expanded the *bra* domain toward the animal pole, both in dorsal and ventral injections (Fig. 2B,D,E,F) and also increased *bra* expression in the organiser, hence the arc normally expressing lower levels of transcripts was undistinguishable from the remaining domain in the MZ (Fig. 2C,D,F). *Hairy2b* MO also expanded the *bra* domain (not shown), but *hairy2a* MO was more effective if the percentages of embryos with *bra* expansions were compared at the same doses (Fig. 2E; Table 1).

**Table 1:** *Bra* expansion at different doses of *hairy2* Mos.

Injection	Doses		
	1.25 ng	2.5 ng	5 ng
Control MO	30% (6/20)	14% (7/42)	22% (14/63)
*hairy2a* MO	56% (15/27)	57% (38/68)	76% (16/21)
*hairy2b* MO	30% (8/27)	44% (28/63)	31% (12/39)

Embryos were injected into one blastomere at the 4-cell stage and *bra* expression was analysed at gastrula stage. The results of two to four experiments were collected and represent the percentage of injected embryos that showed an expanded *bra* domain. The ratio between brackets indicates the number of cases with expanded *bra* in relation to the total number of injected embryos. Changes observed with control MO might reflect normal asymmetries of the *bra* domain, since uninjected sibling controls also showed slight asymmetries in a similar proportion.

Since we obtained the best performance of *hairy2b* MO alone with 2.5 ng (Fig. 2E), we analysed the simultaneous knock-down of both homeologs after injection of a mix consisting of 2.5 ng of each MO. This resulted in an expansion of the *bra* domain in 56% of the injected embryos (Fig. 2G). Therefore, the simultaneous knock-down did not increase the effect on *bra* in comparison with 2.5 ng of each MO alone (57% for *hairy2a* MO, 44% for *hairy2b* MO; Fig. 2G) as it would be expected if both homeologs equally contribute to modulate *bra* expression. Moreover, 5 ng of *hairy2a* MO alone resulted in a higher proportion of embryos with *bra* expansions (76%) than that obtained with 5 ng of the mix containing 2.5 ng of each MO in the simultaneous knock-down (56%) (Fig. 2G), indicating that the *hairy2b* MO could not efficiently supplement *hairy2a* MO. These results together support the idea that the effects of *hairy2a* MO on *bra* expression are consistently more potent than those of the *hairy2b* MO, suggesting that normally, *hairy2a* is more relevant than *hairy2b* in restricting the *bra* territory.

Our results indicate that the expression of *hairy2* throughout the NIMZ confines *bra* to the IMZ, preventing its animal expansion, and also restricts *bra* expression in the organiser.

Next, we wondered whether *bra* could affect *hairy2* expression. Since the three *Xenopus laevis bra* genes have nearly identical expression patterns [5] [7]–[8], loss-of-function analysis in this species is more complex. Therefore, to interfere with Bra activator function, we injected *braEnR* mRNA, a dominant-negative *bra* construct classically used to directly repress *bra* target genes, in which the transcription activator domain of Bra was replaced with EnR [13] [55]. The specificity of this kind of constructs to interfere with the function of T-box transcription factors in vivo was carefully demonstrated in previous works in *Xenopus laevis.* When the EnR domain was fused to a particular T-box transcription factor, only the specific wild type protein, but not other members of the family, was able to rescue the phenotypic effect of the antimorphic construct. Moreover, embryos injected with the EnR domain alone were indistinguishable from uninjected controls [13] [56]–[57].

**Figure 3:**
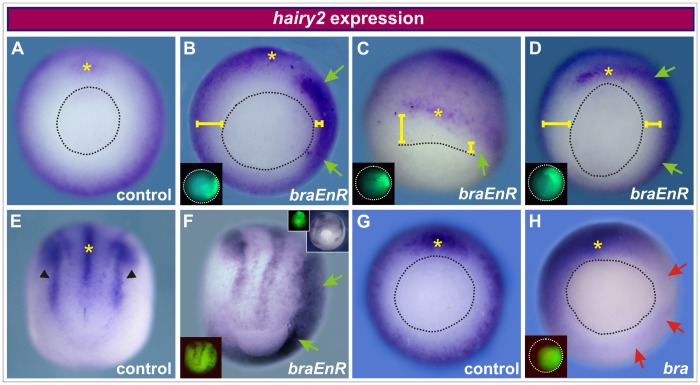
*Bra* restricts *hairy2* expression. Distribution of *hairy2* at gastrula (A–D,G,H) and neural plate stage (E,F). Embryos were injected into a ventral (B,F,H) or dorsal cell (C,D) at the 4-cell stage with 1 ng of *braEnR* mRNA (B–D,F) or with 1 ng of *bra* mRNA (H). DOG fluorescence (left insets) indicates the injected side. The upper right inset in F shows an embryo injected into a dorsal cell at the 4-cell stage with 1 ng of *braEnR* mRNA with its corresponding DOG image at the left. Embryos in B–D, F, and H were fixed when sibling controls as shown in A,E, and G reached stage 11.5, 14, and 10.5, respectively. Embryos are shown in vegetal (A,B,D,G,H), dorsal (C), and posterior-dorsal views (E,F). Yellow asterisks mark the normal expression domain of *hairy2* in the organiser region (A–D,G,H) or in the DML (E). Black arrowheads point to the *hairy2* subdomains demarcating the neural plate border. The black broken line demarcates the blastopore. The green arrows point to ectopic expression of *hairy2.* Comparison of the distance from the blastopore to the vegetal border of the *hairy2* domain between the injected and the non-injected sides (yellow bars) show the vegetalward expansion of *hairy2* expression. Red arrows point to down-regulation of *hairy2*.

We injected 1 ng of *braEnR* mRNA in 1 cell at the 4-cell stage and analysed *hairy2* expression at gastrula and neural plate stages. In gastrulae, *braEnR* increased *hairy2* transcripts and produced a vegetalward expansion of its domain, so that *hairy2*+ cells invaded the ring otherwise corresponding to *bra* in the MZ (78%, n = 35/45), either in ventro-lateral locations after ventral injections (79%, n = 11/14; Fig. 3B) or in dorso-lateral locations after dorsal injections (77%, n = 24/31, Fig. 3C,D). At neural plate stage, when *hairy2* expression is clearly resolved into distinct subdomains, including the neural plate borders and the DML (Fig. 3E), the up-regulation of *hairy2* could be very clearly distinguished in all of the *braEnR*-injected embryos (100%, n = 65). At this stage, ventral injections of *braEnR* resulted in strong ectopic expression of *hairy2* in ventro-lateral locations, including the circumblastoporal collar (100%, n = 36; Fig. 3F), while dorsal injections expanded the DML *hairy2* subdomain (100%, n = 29; right inset in Fig. 3F). Both in injected gastrulae and neurulae the blastopore was more opened in comparison with sibling controls (Fig. 3A–F).

We also performed gain-of-function experiments by injecting *bra* mRNA. In contrast to the interference with *bra* function, *bra* overexpression drastically repressed *hairy2* (80% n = 25/31; Fig. 3H).

Since *bra* overexpression and *braEnR* gave opposite results on *hairy2* expression, we conclude that *bra* is necessary to maintain *hairy2* repressed in the *bra* territory, preventing the vegetal expansion of *hairy2* throughout the entire MZ and the ulterior expansion of the *hairy2* domains in ventro-lateral and DML locations. In conclusion, our results indicate that *hairy2* and *bra* reciprocally confine their normal domains through an antagonistic regulation.

### Inhibition of *bra* Function, Like *hairy2a* Overexpression, Induces Ectopic Dorsal Markers and Impairs Morphogenetic Movements

**Figure 4:**
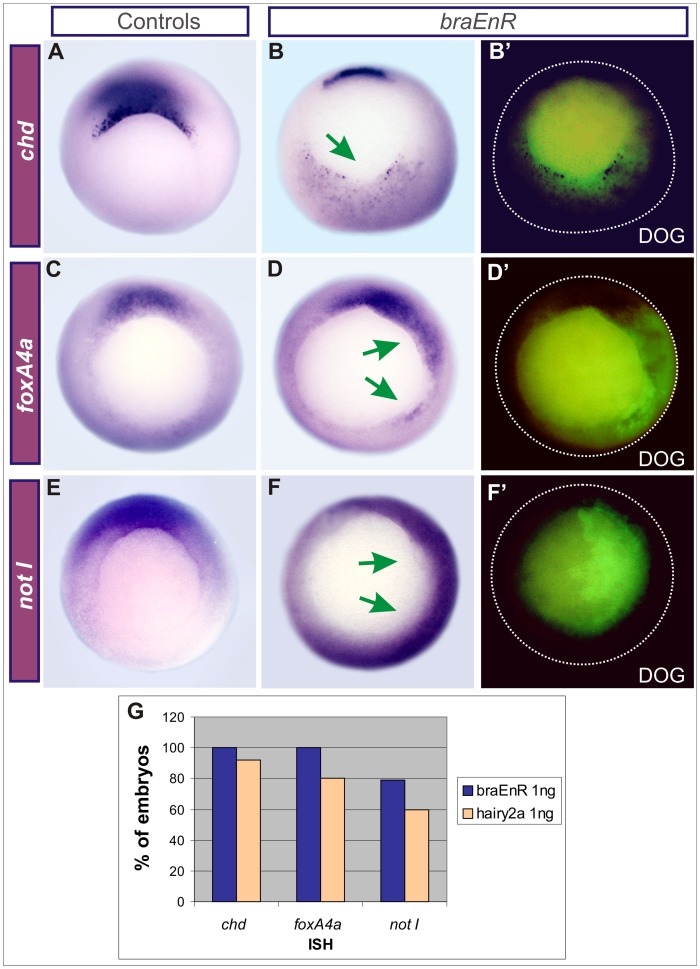
Ectopic induction of DML markers after interfering with *bra* function or overexpressing *hairy2a*. (A,C,E) Uninjected sibling controls. Embryos were ventrally injected into one cell at the 4-cell stage with 1 ng of *braEnR* (B,B’,D,D’,F,F’,G) or of *hairy2a* mRNAs (G). Expression of *chd* (A,B,G), *foxA4a* (C,D,G) and *not I* (E–G) at stage 10.5. The injected side was detected by DOG fluorescence (B’,D’,F’). Green arrows point to the ectopic expression of the analysed markers. At this stage, the markers analysed are normally expressed only in the organiser [63] [112]–[113]. (G) Percentage of gastrulae ventrally injected with *braEnR* (blue bars) or *hairy2a* mRNAs (light orange bars) expressing ectopic *chd*, *foxA4a*, and *not I* (*chd*: 100%, n = 26; *foxA4a*: 100%, n = 14; *not* I: 79%, n = 11/14, for *braEnR*; *chd*: 92%, n = 23/25; *foxA4a*: 80%, n = 8/10; *not I*: 60%, n = 3/5, for *hairy2a*).

Since *braEnR* expanded *hairy2* in the ventrolateral marginal zone (VLMZ), and we previously showed that *hairy2a* overexpression induces ventral expression of DML markers [31], we wondered whether the latter effects could be reproduced by *braEnR*. Remarkably, ventrolateral injections of *braEnR* mRNA induced ectopic *chd*, *foxA4a* and *not I* in the VLMZ in similar proportions than those obtained with *hairy2a* mRNA (Fig. 4).

**Figure 5:**
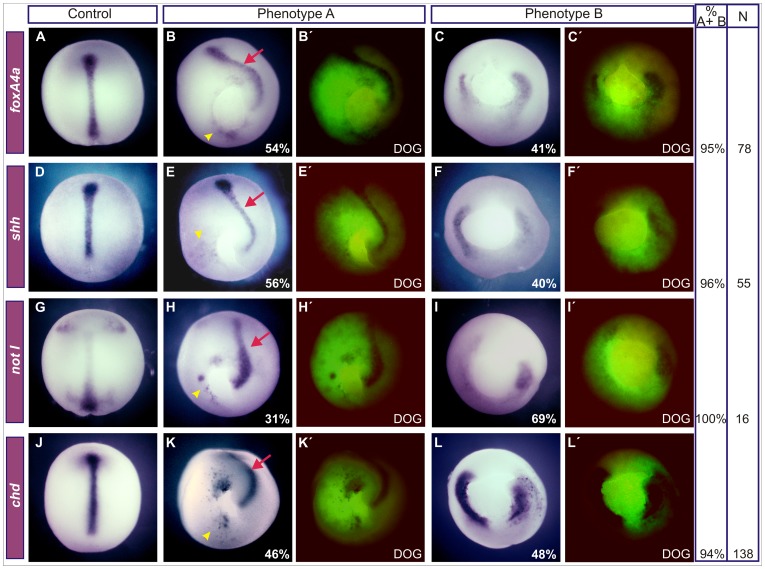
Effects of *braEnR* on morphogenesis and on DML markers at neurula stage. Ventral injections in one cell at the 4-cell stage with 1 ng of *braEnR* mRNA +10 ng of DOG (B–C’,E–F’,H–I’,K–L’). Expression of *foxA4a* (A–C), *shh* (D–F), *not I* (G–I), and *chd* (J–L), when control embryos reached stage 14 (A,D,G,J). At this stage, these markers are normally expressed in the DML [63] [112]–[114]. Arrows point to the dorsal axis. Yellow arrowheads point to cells expressing ectopic DML markers. Injected embryos are shown with the corresponding fluorescence image at the right (DOG) to identify the descendants of the injected cells. Embryos were classified into phenotypes A and B according to the grade of anterior-posterior extension of the dorsal axis. Embryos are shown in dorsal (A,D,G,J), posterior-dorsal (B,E,H) or posterior (C,F,I,K,L) views. The percentages of embryos displaying each phenotype are indicated in B,C,E,F,H,I,K,L. For each marker, % A+B indicates the percentage of affected embryos (A plus B phenotypes) and N, the total number of embryos analysed.

At neurula stage, the severity of the alterations with *braEnR* depended on the distribution of the injected mRNA, as revealed by the tracer. According to the anterior-posterior extension of the dorsal axis, embryos were classified into two phenotypes. In the less severe one (phenotype A; Fig. 5), cells expressing ectopic DML markers remained arrested around the blastopore, which was unable to complete its closure. Since *bra* function was barely impaired in the dorsal region in these embryos, they tended to develop a normal dorsal axis. The phenotype B (Fig. 5) is more severe, since *braEnR* mRNA was widely distributed, thus affecting also the dorsal side. Both the primary axial cells and those expressing ectopic DML markers remained close to the blastopore. Both phenotypes were remarkably similar to those obtained at neurula stages after overexpression of *hairy2a* [31].

In conclusion, the repression of *bra*-target genes or gain of function of *hairy2a* equally promoted the ectopic expression of DML genes in the VLMZ and altered the morphogenetic movements during gastrulation, thus affecting the involution of cells with dorsal axial characteristics.

### Impairing of *bra* Function, as well as *hairy2a* Overexpression, Produces Opposite Effects on *chd*, Depending on the Dorsal-ventral Context

**Figure 6:**
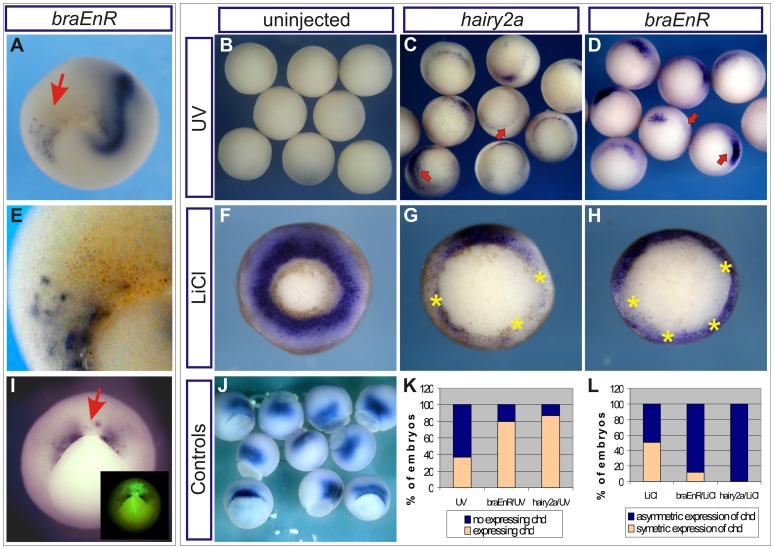
Opposite regulation of *chd* by *hairy2a* or *braEnR* depending on the D –**V context.** Ventral (A,E) or dorsal (I) injections of 1 ng of *braEnR* mRNA into one cell at the 4-cell stage. E corresponds to a high-power view of the embryo shown in A. Embryos were fixed when sibling controls reached stage 14 (A) or 11 (I). Red arrows in A,I indicate the descendants of the injected cells. In A, we made use of the myc-tag epitopes fused to the *braEnR* construct to reveal the distribution of the encoded recombinant protein (brown dots). The injections in I included 10 ng of DOG (inset). Embryos are shown in posterior (A), vegetal (B–D,F–I) or vegetal and dorsal (J) views. (B–D,F–H,J–L) Analysis of *chd* expression when sibling controls (J) reached stage 11 in ventralised (B–D) or dorsalised (F–H) embryos, which were left uninjected (B,F) or were injected with 1 ng of *hairy2a* (C,G,K,L) or of *braEnR* mRNAs (D,H,K,L) before the first cleavage. Yellow asterisks mark the gaps of *chd*-negative cells. (K) Percentage of embryos expressing (light orange bars) or not expressing (blue bars) *chd*. UV-irradiated, uninjected embryos (UV) showed complete loss (67%) or very weak expression (37%) of *chd* (n = 88). *Chd* was expressed in 80% or 87% of UV-irradiated embryos injected with 1 ng of *braEnR* (*braEnR/*UV, n = 55) or of *hairy2a* mRNAs (*hairy2a/*UV, n = 96), respectively. (L) Percentage of embryos with asymmetric (blue bars) or radially symmetric *chd* expression (light orange bars) around the blastopore. The maximum effect of LiCl alone was found in 50% of embryos, which showed complete radial *chd* expression (LiCl, n = 53). *Chd* expression decreased in 88% or 100% of dorsalised embryos that were injected with 1 ng of *braEnR* (*braEnR*/LiCl; n = 41) or of *hairy2a* mRNAs (*hairy2a*/LiCl, n = 23), respectively.

It was interesting to notice that in the ventral region of embryos injected with *braEnR*, the patch of cells in which *bra* activity was blocked (green fluorescent cells in Fig. 5K,K’; brown+ cells in Fig. 6A,E) did not express *chd,* indicating that these cells were unable to ectopically activate *chd*. This marker was mainly activated in the vicinity of those cells, resembling the effects of *hairy2a* (not shown) or *hairy2b* overexpression [34]. These results suggest that ventral cells in which *bra* target genes were repressed with *braEnR* or in which *hairy2* was activated secrete a signal that, in turn, induces *chd* in neighbouring cells. While in the ventral side, both *braEnR* and *hairy2* overexpression were able to induce ectopic *chd* expression, on the dorsal side, *chd* was repressed, and this occurred in the same cells where *bra* was blocked or in which *hairy2* was overexpresed (100%, n = 8 for *braEnR,* Fig. 6I; 83%, n = 12 for *hairy2a,* not shown), in agreement with previous results of *hairy2a* or *hairy2b* overexpression [31] [34].

We made use of UV-irradiation or LiCl-treatment to confirm whether impairing of *bra* function or overexpression of *hairy2a* activate the dorsal marker *chd* in a ventral context but repress it in a dorsal one. UV-irradiation of fertilised eggs destabilises maternal β-catenin, producing embryos with ventral character. LiCl stabilises β-catenin, resulting in completely dorsalised embryos [39].

*Chd* was drastically down-regulated in UV-treated, uninjected embryos (Fig. 6B,K), but when they were injected with *hairy2a* or *braEnR* mRNAs at the 1-cell stage, *chd* transcripts appeared around the entire MZ (Fig. 6C,D,K). In spite of the increase of *chd*, these embryos showed a blastopore more open in relation to sibling controls (Fig. 6J). Therefore, *chd* was induced in ventralised embryos in which *bra*-target genes were repressed or in which *hairy2a* was overexpressed, but those *chd*+ cells could not involute like normal dorsal cells.

In LiCl-dorsalised embryos, *chd* expression was totally radial and symmetric throughout the MZ (Fig. 6F,L). *Chd* expression evidently decreased in dorsalised embryos that were injected with *hairy2a* or *braEnR* mRNAs at the 1-cell stage. Radial expression was lost in these embryos, which showed gaps of *chd*-negative cells and a blastopore noticeably more open in relation to dorsalised, uninjected embryos (Fig. 6G,H,L). These results confirm that in a dorsal context, repressing *bra*-target genes as well as overexpressing *hairy2a* inhibited *chd* and disturbed the morphogenetic movements of dorsal cells.

### An Intact *bra* Function Prevents the Induction of Spurious Incomplete Axes

**Figure 7:**
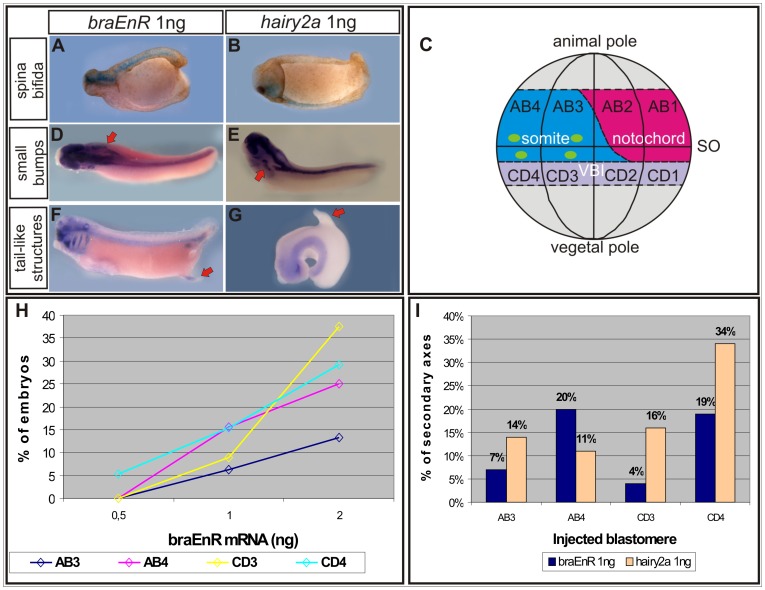
Effects of ventral injections of *braEnR* and *hairy2a* mRNAs. (A,B) Ventral injections into one ventral cell at the 4-cell stage produced spina bifida. (C) Mesodermal fate map of the blastomeres of the 16-cell-stage embryo (modified from [58]). Notochord (pink), somites (light blue), ventral blood islands (VBIs, light violet), Spemann’s organiser (SO). Green dots indicate the injection sites. (D–G) Ventral injections as in C occasionally induced small bumps (arrows, D,E) and tail-like structures (arrows, F,G). Embryos shown in D–G were hybridised with a *sox2* probe. (H) Percentage of embryos with secondary axes+protuberances. Comparison between different doses of *braEnR* mRNA injected into the AB3 (blue), AB4 (pink), CD3 (yellow) or CD4 (light blue) blastomere. (I) Percentage of embryos with secondary axes classified according to the injected blastomere. The bars correspond to *braEnR* (blue) or *hairy2a* (light orange) mRNAs injections. Injections included *nuc-lacZ* mRNA (A,B) or DOG (D–G) as lineage tracers. Embryos were fixed when sibling controls reached stage 28 and are shown in dorsal (A,B,D,G), dorso-lateral (E) or lateral (F) views. The amounts of mRNA injected are indicated in each figure.

**Figure 8:**
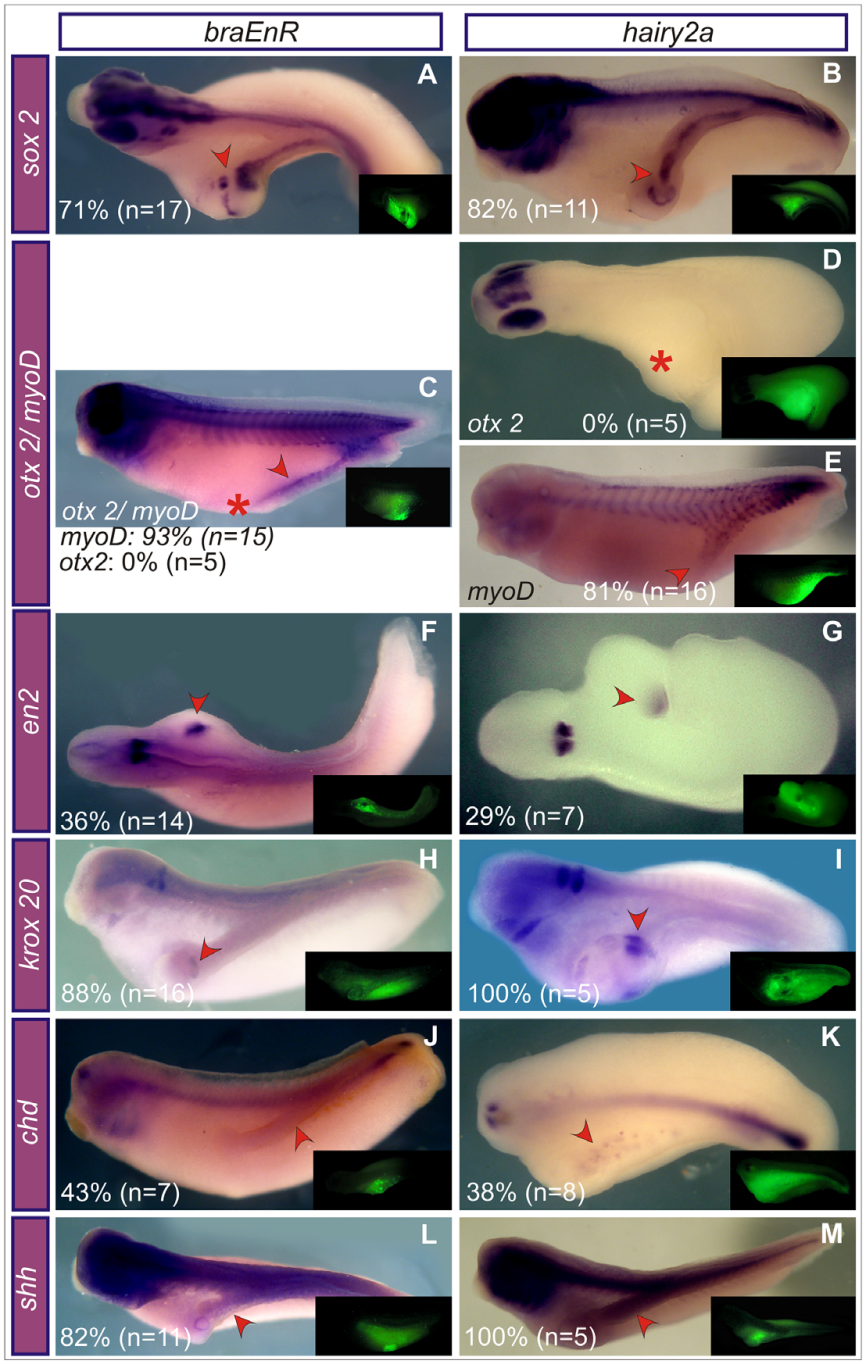
Characterisation of the secondary axes induced by *braEnR* or *hairy2a*. A–M: Ventral injections into one cell at the 16-cell stage with 1 ng of *braEnR* (A,C,F,H,J,L) or of *hairy2a* mRNAs (B,D,E,G,I,K,M). Expression of *sox2* (A,B), *otx2* (C,D), *myoD* (C,E), *en2* (F,G), *krox20* (H,I), *chd* (J,K), and *shh* (L,M) at stage 28. For *shh*, embryos were overstained to preserve color during histological procedures. The injected side was detected by DOG fluorescence (insets). Red arrowheads point to the expression of different markers in the secondary axes. The red asterisk in C,D indicates the absence of *otx2* expression in the secondary axis. The frequency for each marker is shown as percentage of the total number (n) of secondary axes analysed with the corresponding probe. *Sox2* is a general neural marker [115]. *Otx2* is normally expressed in the eyes and forebrain [116]. *MyoD* marks the somitic mesoderm [117]–[118]; *en2*, the midbrain-hindbrain boundary [119]; *krox20*, the third and fifth rhombomeres [120]; and *chd* and *shh*, the DML (see text for details). Embryos are shown with the primary axis in dorsal (A,D,F,G,K), dorso-lateral (L,M) or lateral (B,C,E,H,I,J) views.

Most of the embryos injected into one cell at the 4-cell stage with either *braEnR* or *hairy2* mRNA failed to complete gastrulation and showed generalised disturbances at tailbud stages (Fig. 7A,B). Thus, we performed more localised injections at the 16-cell stage into one ventral blastomere normally contributing to the posterior region of the embryo, but not to the notochord (Fig. 7C) [58]. We obtained dose-dependent phenotypes ranging from spina bifida to protuberances, notably including evident secondary axes without heads. Occasionally, tail-like structures were found (Fig. 7, Fig. 8, and Table 2). The most ventral blastomeres contributed in a greater proportion to the secondary axes (Fig. 7I, Table 2), which contained nervous system, since they expressed the neural markers *sox2* (Fig. 8A,B) and *nrp1* [59] (*hairy2a* 100%, n = 5; not shown). The most anterior marker *otx2* was absent, indicating the lack of forebrain (Fig. 8C,D). Some of them contained midbrain cells (Fig. 8F,G), while most developed hindbrain structures (Fig. 8H,I). We observed a relatively well organised secondary trunk with *myoD*+ cells arranged in somites (Fig. 8C,E) and some superficial, scattered *chd*+ cells, apparently not organised in a notochordal rod (Fig. 8J,K). Sonic hedgehog (*shh*) was detected in the secondary axes (Fig. 8L,M). Therefore, the ectopic axes induced by interfering with *bra* function closely resemble those obtained by *hairy2a* overexpression.

**Table 2:** Effects of ventral injections of *braEnR* or *hairy2a* mRNAs at tailbud stages.

mRNA	Injection site(1 blastomere at 16-cell stage)	Incomplete secondary axes	Spina bifida	Protuberances^(1)^	Incomplete secondary axes+Protuberances	Affected embryos^(2)^	n^(3)^
*braEnR* 0.5 ng	AB3,AB4,CD3 or CD4**^(4)^**	0 (0%)	1 (11%)	0 (0%)	0 (0%)	1 (11%)	9
	AB3	0 (0%)	0 (0%)	0 (0%)	0 (0%)	0 (0%)	16
	AB4	0 (0%)	1 (6%)	0 (0%)	0 (0%)	1 (6%)	17
	CD3	0 (0%)	10 (34%)	0 (0%)	0 (0%)	10 (34%)	29
	CD4	1 (5%)	3 (16%)	0 (0%)	1 (5%)	4 (21%)	19
*braEnR* 1 ng	AB3,AB4,CD3 or CD4**^(4)^**	8 (5%)	24 (16%)	0 (0%)	8 (5%)	32 (22%)	146
	AB3	4 (4%)	29 (26%)	3 (3%)	7 (6%)	36 (32%)	113
	AB4	3 (4%)	17 (20%)	10 (12%)	13 (15%)	30 (36%)	84
	CD3	11 (8%)	25 (17%)	2 (1%)	13 (9%)	38 (26%)	146
	CD4	17 (14%)	9 (7%)	2 (2%)	19 (15%)	28 (23%)	123
*braEnR* 2 ng	AB3	1 (7%)	5 (33%)	1 (7%)	2 (13%)	7 (46%)	15
	AB4	8 (20%)	5 (13%)	2 (5%)	10 (25%)	15 (38%)	40
	CD3	1 (4%)	3 (13%)	8 (33%)	9 (38%)	12 (50%)	24
	CD4	15 (19%)	5 (6%)	8 (10%)	23 (29%)	28 (35%)	79
*hairy2a* 0.5 ng	AB3,AB4,CD3 or CD4**^(4)^**	1 (7%)	5 (33%)	0 (0%)	1 (7%)	6 (40%)	15
*hairy2a* 1 ng	AB3,AB4,CD3 or CD4**^(4)^**	29 (32%)	26 (29%)	0 (0%)	29 (32%)	55 (61%)	90
	AB3	5 (14%)	3 (9%)	0 (0%)	5 (14%)	8 (23%)	35
	AB4	4 (11%)	0 (0%)	0 (0%)	4 (11%)	4 (11%)	36
	CD3	5 (16%)	1 (3%)	3 (9%)	8 (25%)	9 (28%)	32
	CD4	13 (34%)	0 (0%)	4 (11%)	17 (45%)	17(45%)	38

The results from two to four experiments were collected. Each column displays the number of embryos with the indicated phenotype and the percentage between brackets. (1) Protuberances include small bumps that, when analysed with a *sox2* probe, were found to be weak secondary axes (Fig. 7D,E), and tail-like structures, which sometimes were observed (Fig. 7F,G). (2) Sum of all the observed phenotypes: incomplete secondary axes + protuberances + spina bifida. (3) Total number of injected embryos. (4) Injections were performed in any of these four blastomeres, at random.

**Figure 9:**
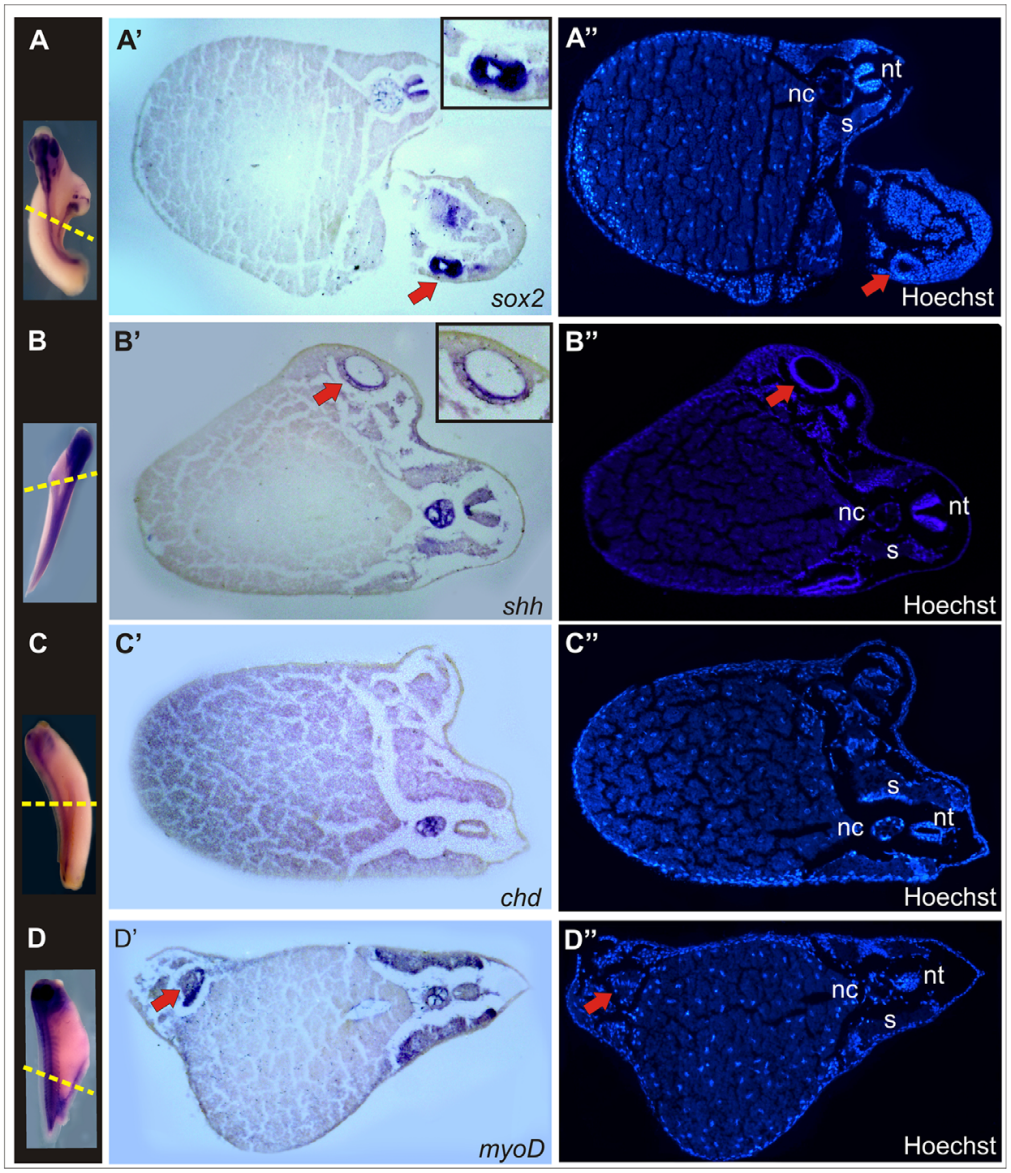
Histological analysis of secondary axes induced by *braEnR*. Expression of *sox2* (A,A’), *shh* (B,B’), *chd* (C,C’) or *myoD* (D,D’). A’,A’’;B’,B’’;C’,C’’; D’,D’’ are transverse sections of the embryos shown in A,B,C,D, respectively. The yellow broken lines show the plane of the sections. (A’’,B’’,C’’,D’’) Nuclear Hoescht staining. The insets in A’ and B’ correspond to high-power views of the secondary neural tubes. Red arrows point to the secondary neural tube (A’,A’’,B’,B’’) or to the somites (D’,D’’) in the secondary axes. nc: notochord; nt: neural tube; s: somites.

Histological analysis of *braEnR*-injected embryos revealed that *sox2* transcripts were located in the germinal neuroepithelium of the primary neural tube, but throughout all layers of the secondary one (Fig. 9A–A’’), which also showed s*hh* expression throughout its ventral half, indicating the presence of an expanded FP (Fig. 9B–B’’). Strikingly, no *shh*+ cells were detected ventrally to the FP, consistent with the observation that *chd*+ cells failed to organise a notochord and that the dorsal mesodermal midline was instead occupied by *myoD*+ somitic structures (Fig. 9C–D’’).

**Figure 10:**
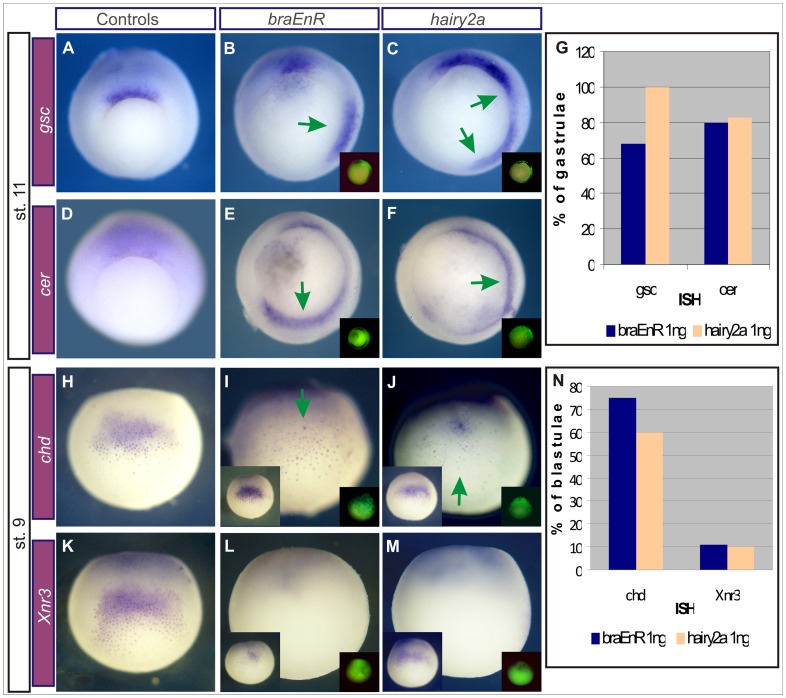
*BraEnR* or *hairy2a* ventrally induce *gsc* and *cer*, but cannot consistently activate ectopic BCNE markers. Ventral injections into one cell at the 4-cell stage with 1 ng of *braEnR* (B,E,I,L) or of *hairy2a* mRNAs (C,F,J,M). (A,D,H,K) Sibling controls. Expression of *gsc* (A–C) and *cer* (D–F) at stage 11. Expression of *chd* (H–J) and *Xnr3* (K–M) at stage 9. Embryos are shown in vegetal (A–F), dorsal (H,K) or ventral views (I,J,L,M). Left insets in I,J,L,M are dorsal views of the embryos shown in the corresponding photography. The injected side was detected by DOG fluorescence (right insets). The green arrows point to the ectopic expression of the markers analysed. (G) Percentage of gastrulae ventrally injected with *braEnR* (blue bars) or *hairy2a* mRNAs (light orange bars) expressing ectopic *gsc*, and *cer* (*gsc*: 68%, n = 40/59; *cer*: 80%, n = 25, for *braEnR*; *gsc:* 100%, n = 25; *cer:* 83%, n = 30, for *hairy2a*). (N) Percentage of blastulae ventrally injected with *braEnR* mRNA (blue bars) or *hairy2a* mRNA (light orange bars), expressing ectopic *chd* or *Xnr3* (*chd:* 75%, n = 23; *Xnr3*∶11%, n = 26, for *braEnR; chd:* 60%, n = 23; *Xnr3∶*10%, n = 51, for *hairy2a*).

At late gastrula, the PM cells express the transcriptional repressor goosecoid (*gsc*), when they have already segregated from the chordal population [60]–[62]. Ventral injections of *braEnR* or *hairy2a* mRNAs induced ectopic *gsc* in the VLMZ at stage 11 (Fig. 10B,C,G). Since *chd* is also normally expressed in the PM [63], the scattered *chd*+ cells observed in some secondary axes might be of PM characteristics, since the secondary axes failed to develop an organised notochordal rod (see Discussion).

A transcriptional cascade triggered by maternal β-catenin in the dorsal side is necessary to establish the complete embryonic axis [40] [64]. An early output of this pathway is the blastula Chordin- and Noggin-expressing center (BCNE), which is important for the formation of the head and appears when zygotic transcription begins [65]. During gastrulation, secreted Cerberus (Cer) from the anterior mesendoderm is necessary to fulfil head development. When ventrally overexpressed, Cer induces secondary heads without trunk-tail structures [61] [66].

Ventral injections of *braEnR* or *hairy2a* mRNAs into one cell at the 4-cell stage induced *cer* expression in the gastrula VLMZ (Fig. 10E,F,G), indicating that the absence of the anterior brain is unlikely attributable to the lack of Cer signalling. We wondered whether the injections were able to induce an ectopic BCNE at late blastula. This center contains the precursors of the forebrain and the SO and expresses the neural inducers *chd* and nodal-related 3 (*Xnr3*) [65] [67]–[69]. While *chd* was detected in the ventral side, albeit in much fewer cells than in the endogenous center, ectopic *Xnr3* was rarely and faintly detected (Fig 10I,J,L–N). In conclusion, although repressing *bra* target genes or an excess of *hairy2a* activity induced ventral expression of the head inducer *cer*, this was not preceded by a consistent activation of BCNE markers.

### *Hairy2a* Overexpression, Like Ventral Inhibition of *bra* Function, Induces Cell Death in the Posterior Region

It was reported that ventral injections of *braEnR* promoted apoptosis in the posterior region since neurula stage [27]. When we injected the same construct at the 16-cell stage, the maximum frequency of secondary axes was around 20%, but they were very rarely observed in embryos ventrally injected with *braEnR* or *hairy2a* mRNAs into one cell at the 4-cell stage, which showed severe disturbances. Thus, we wondered whether cell death could explain the variability of the frequencies. To answer this question, we compared cell death between sibling embryos injected with *braEnR* mRNA or *hairy2a* mRNA into one ventral cell at the 4-cell stage, or into the most ventral-vegetal blastomere (CD4) at the 16-cell stage.

**Figure 11:**
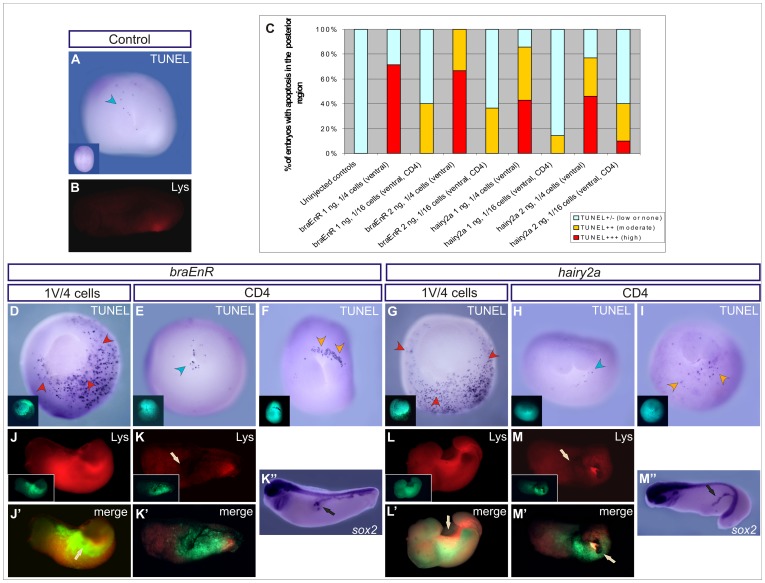
Apoptosis in embryos injected with *braEnR* or *hairy2a* mRNAs occurs in the posterior region. Embryos were injected with 1 or 2 ng of *braEnR* (C,J–K’’ and C,D–F, respectively) or with 1 or 2 ng of *hairy2a* mRNAs (C,L,M’’ and C,G–I, respectively) into one ventral cell at the 4-cell stage (1V/4 cells, C,D,G,J,J’,L,L’) or into the CD4 blastomere at the 16-cell stage (C,E,F,H,I,K–K’’,M–M’’). (A,B) uninjected sibling controls. Left inset in A corresponds to the dorsal view of the same embryo. TUNEL assays (A,C,D–I) or Lys staining (B,J–K’,L–M’) were performed at stage 14 or 28, respectively. (K’’,M’’) show ISH of *sox2* in the same embryos shown in K,M, respectively, to reveal the secondary axis. Turquoise, orange and red arrowheads point to low, moderate or high levels of apoptosis, respectively. Left insets in D–I,J,K,L,M show the injected side revealed by DOG fluorescence. Arrows in K,K’’,M,M’’ point to secondary axes; white arrows in J’,L’ point to spina bifida, and in M’, to the unclosed blastopore. (J’,K’,L’,M’) merge of images shown in J,K,L,M, respectively. Embryos are shown in posterior (A,D–I) or lateral (B,J–M’’) views.

The TUNEL assay detects the 3′-OH ends generated by the DNA fragmentation occurring during apoptosis [70]. We performed the TUNEL assay at stage 14 and classified the embryos into three categories, according to the appearance of TUNEL+ cells in the posterior region at the neural plate stage, as follows: TUNEL+/− (none or low), TUNEL++ (moderate), and TUNEL+++ (high) (Fig. 11A,C,D–I, Table 3). Apoptotic cells in controls were found in the anterior neural plate, but the posterior region showed none or a few TUNEL+ cells (Fig. 11A and light blue bars in Fic. 11C), as previously reported [71]. Only embryos injected with either mRNA into one ventral cell at the 4-cell stage and one embryo injected with the highest amount (2 ng) of *hairy2a* mRNA into the CD4 blastomere at the 16-cell stage (10%, n = 10) showed high levels of apoptosis (TUNEL+++) in the posterior region (red bars in Fig. 11C; Fig. 11D,G; Table 3), and the dying cells were found around the unclosed blastopore. In comparison with the embryos ventrally injected at the 4-cell stage, apoptosis notably decreased in embryos injected with either mRNA into the CD4 blastomere at the 16-cell stage. Some of these embryos were indistinguishable from controls (light blue bars in Fig. 11C; Fig. 11E,H; Table 3) in terms of TUNEL staining, others presented moderate levels of apoptosis in the posterior region (orange bars in Fig. 11C; Fig. 11F,I; Table 3), and except for the aforementioned embryo injected with 2 ng of *hairy2a* mRNA, they never fell within the TUNEL+++ category (Fig. 11C, Table 3).

**Table 3:** TUNEL analysis in embryos injected with *braEnR* or *hairy2a* mRNAs.

Injection	TUNEL+/−(low or none)	TUNEL++(moderate)	TUNEL+++(high)
Uninjected controls	100% (11/11)	0% (0/11)	0% (0/11)
*braEnR* 1 ng, 1/4 cells(ventral)	29% (4/14)	0% (0/14)	71% (10/14)
*braEnR* 1 ng, 1/16 cells(ventral, CD4)	60% (6/10)	40% (4/10)	0% (0/10)
*braEnR* 2 ng, 1/4 cells(ventral)	0% (0/12)	33% (4/12)	67% (8/12)
*braEnR* 2 ng, 1/16 cells(ventral, CD4)	64% (7/11)	36% (4/11)	0% (0/11)
*hairy2a* 1 ng, 1/4 cells(ventral)	14% (1/7)	43% (3/7)	43% (3/7)
*hairy2a* 1 ng, 1/16 cells(ventral, CD4)	86% (6/7)	14% (1/7)	0% (0/7)
*hairy2a* 2 ng, 1/4 cells(ventral)	23% (3/13)	31% (4/13)	46% (6/13)
*hairy2a* 2 ng, 1/16 cells(ventral, CD4)	60% (6/10)	30% (3/10)	10% (1/10)

Sibling embryos were injected with 1 or 2 ng of *braEnR* mRNA or with 1 or 2 ng of *hairy2a* mRNA into 1 ventral cell at the 4-cell stage, or into the most ventral-vegetal blastomere (CD4) at the 16-cell stage, or were left uninjected (controls). At neural plate stage, embryos were fixed and processed for TUNEL analysis, and were classified into three categories, according to the appearance of TUNEL+ cells in the posterior region, as follows: TUNEL+/− (low or none), TUNEL++ (moderate), and TUNEL+++ (high).

The fluorescent dye Lys accumulates in lysosomes and was previously used as indicator of cell death [54] [72]. At stage 28, faint Lys staining was scattered in control embryos, with low staining in the posterior region (Fig. 11B). Although embryos injected with *braEnR* or *hairy2a* mRNA into the CD4 blastomere developed a secondary axis, they were indistinguishable from uninjected controls in terms of Lys staining (93%, n = 15 for *braEnR*; 100%, n = 14 for *hairy2a*; Fig. 11K–K’’, M–M’’). In contrast, very strong staining was detected in the most posterior region of embryos injected with either mRNA in one ventral cell at the 4-cell stage, and they exhibited evident morphogenetic distortions (88%, n = 8 for *braEnR*; 100%, n = 9 for *hairy2a*; Fig. 11J,J’,L,L’). Moreover, Lys staining partially co-located with DOG fluorescence (Fig. 11J’,L’), indicating that not only the cells in which *bra* target genes were repressed or *hairy2a* was overexpressed were dying, but they could also induce cell death in neighbouring tissues. Notably, the secondary axes induced in CD4-injected embryos were observed in a region without appreciable cell death (arrows, Fig. 11K,K’’,M,M’’).

Therefore, the increase in cell death and the appearance of severe disturbances is a feasible explanation for the variable incidence of secondary axes in our experiments. In conclusion, *bra* should be fully functional and *hairy2* should be maintained in low activity in the ventral half of the embryo for the survival of the posterior cells.

### Bra Prevents Ectopic Dorsal Induction by Preventing *hairy2* Derepression

**Figure 12:**
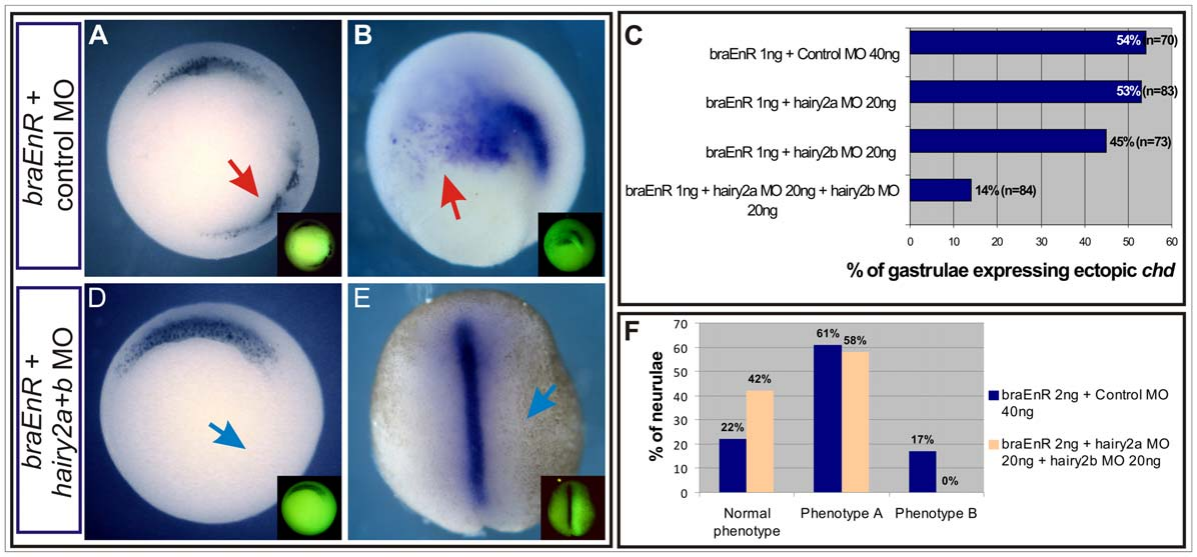
The effects of *braEnR* are mediated by *hairy2.* A–F: Ventral injections in one cell at the 4-cell stage with *braEnR* mRNA plus the following MOs: control MO (A–C,F), *hairy2a* MO (C), *hairy2b* MO (C), or *hairy2a+b* MOs (C–F). Expression of *chd* at stage 10.5 (A,C,D) or stage 14 (B,E,F). The doses of injection are indicated in C,F. The injected side was determined by DOG fluorescence (insets). Red arrows point to the ectopic *chd*+ cells. Blue arrows indicate the location of the injection in rescued embryos. Embryos are shown in vegetal (A,D), posterior-dorsal (B) or dorsal (E) views. (C) Percentage of injected gastrulae expressing ectopic *chd* in the VLMZ. n: total number of injected embryos. (F) Percentages of injected neurulae showing normal morphology and *chd* expression (Normal phenotype), or Phenotype A or B (as in Fig. 4). Blue bars: embryos injected with *braEnR* mRNA plus control MO (n = 36). Light orange bars: embryos injected with *braEnR* mRNA plus *hairy2a+b* MOs (n = 31).

Since *braEnR* and *hairy2a* overexpression gave identical results, we investigated whether the phenotypes observed when we blocked *bra* function were specifically produced by the derepression of *hairy2.* When *braEnR* mRNA was co-injected with either *hairy2* MO separately into one ventral cell at the 4-cell stage, there was not a significant change in the proportion of gastrulae with ectopic *chd* in comparison with *braEnR* mRNA+control MO co-injections (Fig. 12A,C). Notably, when *braEnR* mRNA was co-injected with *hairy2a+b* MOs into one ventral cell at the 4-cell stage, most of the embryos did not reveal ectopic *chd* (Fig. 12D,C). In neurulae, after co-injection of *braEnR* mRNA with *hairy2a+b* MOs, the proportion of unaffected embryos increased nearly two-fold in comparison with *braEnR* mRNA+control MO co-injections (Fig. 12F), while the most severe phenotype (phenotype B, Fig. 12B) completely disappeared (Fig. 12F). Embryos with rescued phenotypes did not show ectopic *chd*, achieved a normal elongation of the notochord and completed the blastopore closure (Fig. 12E,F). In conclusion, an intact *bra* function is necessary to maintain both homeologs of *hairy2* repressed in the VLMZ in order to prevent the ectopic expression of DML genes in this region and also contributes to keep normal morphogenetic movements during gastrulation.

## Discussion

Based on the dynamics of their expression and on the results of the functional experiments with localised injections, we present evidence for a previously unrecognised role for *bra* in preventing the appearance of trunk duplications by maintaining the dorsal fates repressed in the ventrolateral IMZ of the gastrula, through the exclusion of *hairy2*.

### *Bra* is Necessary to Prevent the Derepression of *hairy2* and the Occurrence of its Inductive Functions in the Ventral Side

The following line of evidence supports that *hairy2* mediates the effects of *bra* interference: a) *hairy2a* and *braEnR* mRNAs injections result in striking similar phenotypes; b) *braEnR* potently induces ectopic *hairy2* expression*;* c) the knock-down of both *hairy2* homeologs significantly rescues the ectopic expression of the dorsal marker *chd* and contributes to restore the normal development of embryos in which *bra* function was interfered.

**Figure 13:**
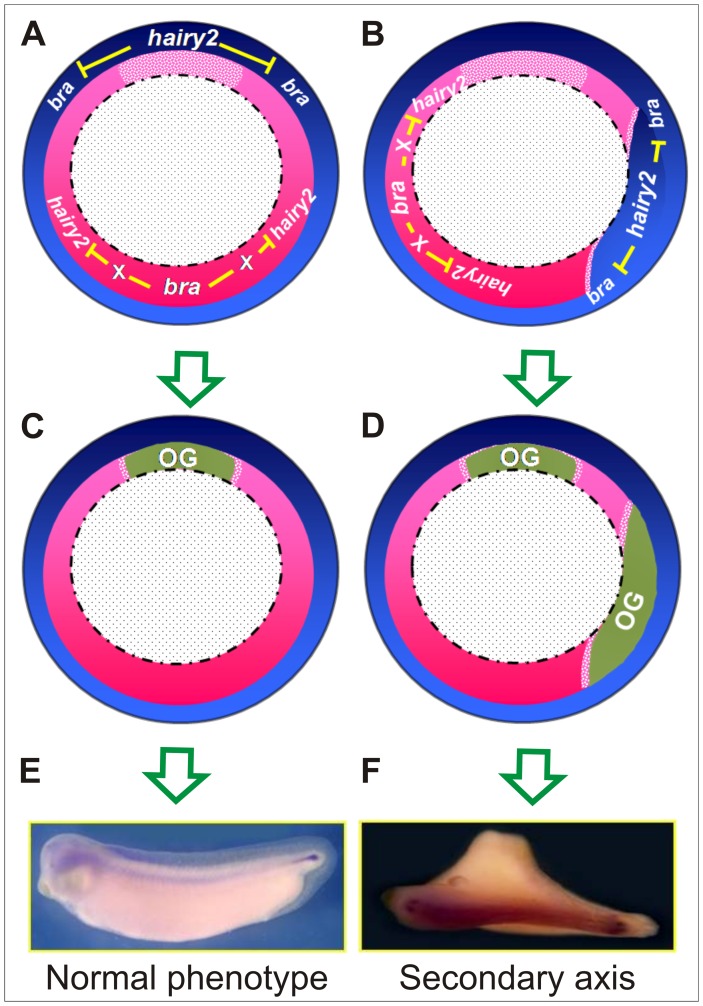
*Bra* maintains the embryonic dorsal-ventral pattern by repressing *hairy2* in the MZ. Diagrams of gastrulae in vegetal views. The black broken line represents the blastopore lip. (A,C,E) In control embryos, *hairy2* is expressed in the NIMZ with maximum levels in the dorsal side (blue gradient), while *bra* is expressed in a more vegetal location, in the IMZ, with highest expression in the ventral region (pink gradient), while a gap of lower expression is observed in the organiser region (light pink). Through mutual antagonism, both genes establish complementary patterns and participate in a regulatory mechanism that ensures the normal development of the embryo (A). Thus, organiser specific genes (OG, green) are expressed only in the dorsal region (C) and the embryogenesis proceeds resulting in a normal larva (E). (B,D,F) However, if this equilibrium is perturbed and *bra* is inhibited in the VLMZ, *hairy2* is derepressed, resulting in a vegetalward displacement of its domain (B), leading to the ectopic activation of OG in the VLMZ (D). This finally results in the establishment of a headless secondary axis without notochord (F). *Bra* should be acting indirectly, through the induction of a repressor of *hairy2* (X).

Studies on the role of *bra* have been mainly focused on posterior mesoderm development, but there is also evidence for the involvement of *bra* in delimiting contiguous territories during *Xenopus* embryogenesis. For example, among the two subpopulations of the axial mesoderm, *bra* inhibits the active cell migration behaviour of the PM and promotes the convergent-extension movements of the notochordal cells [27]–[28]. Within the presumptive non-axial mesoderm, *bra* is expressed in the animal half of the IMZ and restricts the expression of the ventral mesoderm inducer *Xnr2 (Xenopus nodal-related 2)* to the vegetal half [58]. In zebrafish, *bra (ntl)* disfavours medial FP development but promotes the notochordal fate [73]. Now, we provide evidence that *bra* is also necessary to prevent the specification of DML fates within the non-organiser mesoderm field by precluding the vegetal expansion of the *hairy2* domain into the ventrolateral IMZ, thus impeding the appearance of spurious, incomplete axes (Fig. 13).

*Bra* overexpression inhibited *hairy2* in the MZ, while *braEnR* gave the opposite result, indicating that the effect of the antimorphic construct was specific. These results support the hypothesis that during normal development, *bra* is necessary to maintain *hairy2* repressed in the *bra* territory, preventing the vegetal expansion of *hairy2* throughout the entire MZ, since both dorsal and ventral injections of *braEnR* resulted in up-regulation of *hairy2*. Since *bra* is a transcriptional activator [13], the most likely explanation is that *bra* induces a transcriptional repressor that inhibits *hairy2* in the ventrolateral IMZ (X, Fig. 13). A similar model was proposed to explain the repression of *Xnr2* by *bra* on the MZ [74]. Studies in other model organisms like mouse and zebrafish described that Bra (Ntl) binds regulatory regions of several genes, including positive and negative effectors of posterior development [75]–[77]. It will be necessary to elucidate how *bra* is related to *hairy2* in this complex gene network.

*Hairy2* behaves as a cell context factor which predisposes the cells towards a particular decision in response to extrinsic signals during muscle and neural differentiation [78]. Notably, *hairy2* can function as a transcriptional repressor or as a non-transcription factor with inductive properties, depending on the regional context. The latter function, mediated by the C-terminal WRPW tetrapeptide motif, is responsible for the ventral activation of *chd* by *hairy2b* in neighbouring cells [34]. Our results demonstrate that repressing *bra* target genes mimics the effects of *hairy2* overexpression both in dorsal and ventral contexts, and this is explained because *braEnR* was able to induce *hairy2* expression both in dorsal and ventral injections. Therefore, the inductive, non-cell autonomous function of *hairy2a* or *braEnR* on *chd* expression only arises in a ventral context, while in a dorsal one, both manifest a repressive, cell-autonomous behaviour.

All together, these results indicate that *bra* is necessary throughout the non-organiser IMZ to prevent the derepression of *hairy2* and the occurrence of its inductive functions. On the other hand, dorsal injections of either *braEnR* or *hairy2a* result in *chd* repression and similarly impair morphogenetic movements, while *hairy2* MOs are able to rescue these effects of *braEnR*. These results suggest that in the dorsal region, *bra* is necessary to prevent an excess of *hairy2* transcriptional repressor activity, contributing to ensure the progress of morphogenetic movements with a normal balance in the specification of the DML precursors (notochord, FP, PM).

It is worthwhile noting that in mouse chimeric embryos in which embryonic stem cells mutant for the T-gene were injected into wild type blastocyst hosts, the posterior neuropore was unable to close, the mutant cells remained in the surface of the distant tail, and small lumps of tissue on the posterior region, tails with a branch or divided tails, or even a duplicated tail containing somites were observed [79]–[80]. This situation is more comparable than that of homozygous T-mutants to our manipulations in *Xenopus* embryos, where a group of ventral-posterior cells, in which *bra* target genes were repressed, were able to interact with a context with an intact *bra* function. The frog embryo offers the advantage to perform a more spatially controlled manipulation, and we could only obtain stronger and distinguishable secondary axes when we performed more localised injections of the antimorphic Bra in ventral-posterior cells.

### *Hairy2* Contributes to Confine *bra* Expression

There is some evidence that the definitive spatial pattern of *bra* is achieved through the release from repression in those regions where it is required rather than through specific activation [81]. The transcriptional repressors SIP1 (a member of the δEF-1 family) and Gsc (a homeodomain protein) directly bind the *bra* promoter and restrict *bra* expression in two aspects: a) SIP1 prevents its expansion to the other germ layers (ectoderm and endoderm) [22] [82]–[83]; b) Gsc prevents its expansion to the PM, restricting *bra* to the chordamesoderm within the population of DML cells segregating during gastrulation [7] [62] [84]–[86].

However, other factors might contribute to confine *bra*, since proteins that recognise the SIP1 binding sites seem to be functionally relevant only at the beginning of gastrulation, and *bra* was down-regulated in animal cap cells treated with high Activin concentrations even when *gsc* was inhibited [22] [87]. Our previous findings after depleting *hairy2a* suggested that this gene is necessary to restrict *bra* expression within DML precursors [31]. In this work, we extended the analysis to the whole MZ by depleting each *hairy2* homeolog, concluding that *hairy2* participates in both aspects of *bra* confinement. Within the MZ, *hairy2* restricts *bra* to the prospective mesoderm layer in the IMZ, by preventing its expansion toward the prospective ectoderm in the NIMZ.

In the MZ, *hairy2a* seems to be more relevant than *hairy2b*, as the comparison of the morphants and the simultaneous knock-down suggest (Fig. 2). Previously reported knock-down analysis indicates that both homeologs participate in the development of the DML, which expresses similar levels of *hairy2a* and *hairy2b*, but the NCC preferentially require *hairy2b*, which is more abundantly expressed there [30]. Although their relative expression levels in the MZ were not assessed, gain-of-function indicates that both homeologs are able to repress *bra* and to affect NCC in the same way [31]–[32]. Therefore, the local relative abundance of the transcripts might account for the differential MOs effects, perhaps indicating a divergence between homeologs in their spatial regulation rather than in the functional ability of their encoded proteins.

It is interesting to notice that while *hairy2a* appears to be more relevant than *hairy2b* to restrict *bra* expression in the MZ, on the other side of this regulatory network, *bra* prevents the derepression of both *hairy2* homeologs in the VLMZ. This is supported by the observation that the single knock-down of each homeolog is insufficient, but the simultaneous knock-down of *hairy2a* and *hairy2b* is needed to significantly rescue the ectopic expression of DML genes and the perturbation of the gastrulation movements produced by *braEnR* mRNA injections (Fig. 12).

During DML development, the appearance of the *bra* expression gap correlates with the notable increase of *hairy2* in the organiser region. Since dorsal depletion of *hairy2* obliterated that gap, the transient down-regulation of *bra* might be related with the role of *hairy2* in inhibiting the chordamesoderm program in a subset of DML precursors, when the dorsal cells begin to involute, allowing the specification of FP and PM [31] [33]. Indeed, the complementary patterns of both genes persist in the DML structures, since *bra* is only expressed in the chordamesoderm, while *hairy2* transcripts are distributed in the PM and the FP but not in the notochord [5] [31] [36].

HES proteins directly bind to N- or E-box DNA sequences of target promoters [88]. Noteworthy, a fragment of the *Xenopus bra* promoter, sufficient to drive reporter gene expression to the MZ, contains two E-box motifs which are conserved in mouse [22]. It will be interesting to elucidate whether *hairy2* can directly repress *bra* through these sites.

### *Bra* is Involved in a Zygotic Checkpoint that Prevents the Induction of an Incomplete Secondary Axis

The secondary axes that we obtained as a consequence of localised *bra* interference or *hairy2a* overexpression were headless and lacked notochord. They had trunk characteristics and were remarkably similar to those obtained with *hairy2b* overexpression [33].

We presume that the absence of notochord in the secondary axes might be explained because the ectopic dorsal cells adopt FP or PM fates. At stage 11, the patches of ectopic *chd* obtained after injections with *braEnR* or *hairy2a* mRNA in the CD4 blastomere (in the same conditions than those resulting in secondary axes) were similar (80%, n = 10 for *braEnR*; 85%, n = 13 for *hairy2a*; not shown) to those obtained after injections in one ventral cell at the 4-cell stage. Notably, when analysed at stage 28, *chd+* cells were observed in the secondary axes in a lower proportion of the injected embryos and were detected in lower levels in comparison to earlier stages (43%, n = 7 for *braEnR*; 38%, n = 8 for *hairy2a*; Fig. 8J,K), but never formed a notochord, while the majority of the secondary axes were positive for other markers normally expressed in the trunk region, like *shh*, *sox2*, or *myoD* (Fig. 8). Indeed, in embryos injected with *hairy2a* or with *braEnR* mRNAs into one ventral cell at the 4-cell stage, ectopic *chd+* cells were unable to involute, since they remained in the border of the still opened blastopore in neurulae (this work and [31]).

It was previously shown that, among the precursors of the embryonic DML in the Spemann’s organiser, *hairy2* impedes the involution of notochordal cells by promoting FP in *Xenopus* [31] and also favours PM fates at the expense of the notochord [33]. Indeed, injections of either mRNA into one ventral cell at the 4-cell stage (Fig. 10B,C,G) or into the CD4 blastomere (85%, n = 13 for *braEnR*; 64%, n = 14 for *hairy2a*; not shown) resulted in ectopic expression of the PM marker *gsc* at stage 11. Although at this stage we probably still observe a co-ectopic expression of *gsc* and *chd* in the VLMZ corresponding to DML precursors that have not yet completely segregated, they might later be unable to adopt a notochordal fate but preferentially choose the other alternative fates of the DML populations, i.e., the PM or the FP, accounting for the lower levels of *chd+* cells and the lower proportion of secondary axis with *chd+* cells in the injected embryos. This hypothesis is supported by the following evidence: a) the secondary axes that we obtained have a widened FP (Fig. 9). This is supported by the fact that zebrafish mutants for *no tail* (*ntl),* the homolog of *bra,* lack notochord but they contain a wider FP, indicating that *bra* antagonises the development of the FP in favour of the notochord [73] [89]–[90]; b) *chd* is normally expressed in the notochord and in the PM [63], but the notochord is absent from the secondary axes in the *hairy2a-* or *braEnR-*injected embryos. Therefore, the scattered *chd*+ cells that they contain might have migrated haphazardly, according to the active migration behaviour of the PM, which was shown to be induced by *gsc* [91]. The fact that embryos injected with either mRNA at the 4-cell or the 16-cell stage activate ectopic *gsc* expression indicates that they were able to specify cells with PM characteristics.

In addition, in embryos injected with *hairy2a* or with *braEnR* mRNA into one ventral cell at the 4-cell stage, we observed high levels of apoptosis surrounding the unclosed blastopore, where the ectopic *chd+* cells remained arrested. Although embryos injected into the CD4 blastomere showed significantly lower levels of apoptosis in the posterior region, a considerable proportion of them revealed moderate levels of TUNEL staining, when sibling controls presented low or none apoptotic cells in the same region (Fig. 11C,F,I). Therefore, cell death might in part account for the absence or the lower expression of *chd* in the secondary axes, indicating that some *chd+* cells occasionally migrate anteriorly and survive in these conditions.

Among the genes directly activated by maternal β-catenin, only *siamois* and *twin* are able to induce secondary axes containing trunk and head. Both are the earliest genes known to be zygotically activated in the BCNE center, and in turn, they induce other BCNE or organiser factors like *chd, noggin, foxA4a* and *gsc* [61] [69] [92]–[95]. On the other hand, genes that are not direct targets of β-catenin but are later activated in the BCNE or in the organiser, such as *chd* and *gsc,* induce incomplete secondary axis similar to the ones we obtained [63] [96]. These genes were ectopically induced by *braEnR* or *hairy2a* mRNAs at gastrula stage, when the expression of *chd* is normally restricted to the organiser and becomes independent of β-catenin signalling [65]. However, at stage 9, ectopic *chd* was very faintly induced, and neither injection consistently induced ectopic *Xnr3*, which was proposed as a direct target of β-catenin [69] [97]–[98]. Moreover, while ventral injections of β-catenin failed to induce *hairy2* expression [33], *braEnR* strongly activated *hairy2* and besides, promoted *chd* expression in embryos depleted of β-catenin activity by UV treatments.

All this evidence suggests that *bra* is involved in a later zygotic checkpoint, down-stream of the earliest transcription event triggered by maternal β-catenin. Thus, in early embryogenesis, there are two critical times in which dorsal development must be repressed in the ventral side: one, under maternal control, which prevents the accumulation of nuclear β-catenin [41] [43]–[45]; the other one, under zygotic control mechanisms [46], among which we now propose an additional one involving *bra,* through the repression of *hairy2*. It is likely that the mechanism by which *bra* prevents ectopic axis development extends beyond gastrulation, since we have occasionally observed secondary tail-like structures. In *Xenopus*, the tail develops from secondary neurulation as a continuation of events triggered during gastrulation in the late blastopore, and *bra* expression persists in the tailbud [99]–[100].

The complete development of the head requires the early expression of BMP inhibitors in the BCNE center, which predispose the prospective neuroectoderm to form the brain, but it also requires vertical signals from the anterior endoderm during gastrulation, among which Cer is found. Notably, Cer induces secondary heads which do not contain any trunk-tail axial structures such as somites or notochord [61] [65]. We found out that *braEnR*- or *hairy2-*injected embryos ectopically express *cer*, and still they fail to develop head structures in the secondary axes. Since these injections were unable to consistently induce ectopic BMP inhibitors at stage 9, it is tempting to speculate that the ventral ectoderm in our experiments was incompetent to respond to Cer signalling.

Similarly to *braEnR* injections, *hairy2* overexpression induced programmed cell death at neurula stage in the posterior region. Apoptosis is an evolutionary conserved physiological mechanism fundamental to developmental processes, since it sculpts or deletes structures, controls cell number, and eliminates abnormal, misplaced, or nonfunctional cells [101]. Since *hairy2* promotes cell survival in NCC progenitors, where it is normally expressed [102]–[103], the apoptotic effect that we observed might be triggered as a protective mechanism to remove wrongly specified cells. Thus, through the inhibition of *hairy2*, *bra* prevents the activation of dorsal genes in ectopic regions, guaranteeing the survival of cells with ventral-posterior fates. However, if dorsal mis-specified cells happen to escape cell death, they are able to organise an incomplete secondary axis.

Fetus in fetus and parasitic conjoined twins are human pathologies that consist in a defective fetus attached externally, or located within a relatively normal fetus, respectively. The worldwide incidence of the former is about 1∶500,000 births, while the latter occurs with an incidence of 1∶100,000 births. DNA and chromosomal analysis indicates that the host and the defective fetus are genetically identical. The classical fusion theory, based on the pattern of the anomalies but not on embryological models of their pathogenesis, proposes that these pathologies result from the secondary union of two monozygotic embryonic discs [104]–[108]. In spite that the pathogenic mechanisms remain theoretical and are unclear, it is remarkable that the brain is always vestigial or missing in the defective twin fetus, which joins the normal twin at either ventral or dorsal locations [109]. This strikingly resembles the secondary axis without anterior brain that we obtained, which joined the primary axis either dorsally (Fig. 8A,B) or ventrally (Fig. 8C,E). Interestingly, a truncated BMP-4 receptor induces ectopic expression of dorsal genes and secondary axis like those obtained with *braEnR* or *hairy2* [110]–[111], indicating that BMP signalling normally maintains ectopic trunk development suppressed in ventral regions. Thus, although we cannot exclude the fusion theory, failure in control mechanisms resulting in ectopic derepression of dorsal specific genes with the capability to induce a truncated secondary axis might as well contribute to these human pathologies.
